# The neuroscience of human empathy for pleasure: protocol for a scoping review

**DOI:** 10.1186/s13643-024-02481-9

**Published:** 2024-03-02

**Authors:** Manuel Mello, Martina Fusaro, Salvatore Maria Aglioti

**Affiliations:** 1 Sapienza University of Rome and CLN2S@Sapienza, Istituto Italiano Di Tecnologia (IIT), Rome, Italy; 2 Social Neuroscience Lab, Santa Lucia Foundation, Rome, Italy

**Keywords:** Empathy, Pleasure, Vicarious reward, Neural correlates of empathy, Positive emotions

## Abstract

**Objective:**

The neuroscience of human empathy for pleasure and positive affect is an emergent, scarcely addressed topic. The main aim of this scoping review is to map the impact of this new research domain on the field of social and affective neuroscience.

**Introduction:**

Most of the literature on empathy and affect sharing has hitherto focused on negative emotions, with a special focus on pain. However, understanding, sharing, and reacting to others’ pleasures is an evolutionarily and socially important function. Our scoping review addresses this gap in the literature and tries to unify the available information under the empathy for pleasure umbrella.

**Inclusion criteria:**

This scoping review is intended to cover studies on empathy for positive emotions, pleasant sensory outcomes, and other rewards in healthy individuals and neurological/neuropsychiatric/neurodevelopmental disorders populations.

**Methods:**

We will perform a systematic search in the Medline (PubMed), Scopus, and Web of Science (WoS) databases. Two authors will independently screen all titles, abstracts, and then full-text articles that meet the inclusion criteria. The year range of interest will be 2000–2022, and only journal articles published in English will be assessed. Data will be extracted and presented in tables and/or graphical representations to synthesize and describe the results. The extracted data will be reported in a comprehensive summary.

**Results:**

The final manuscript is intended for submission to an indexed journal in late 2023/beginning 2024.

**Conclusions:**

To our knowledge, the present scoping review will be the first to address the variety and heterogeneity of available evidence on human empathy for pleasure. We ultimately aim at perusing the growing literature on this far-reaching field of study and informing future research.

**Systematic review registration:**

The neuroscience of human empathy for pleasure: Protocol for a scoping review. 10.17605/OSF.IO/W7H6J. (December 27, 2022).

**Supplementary Information:**

The online version contains supplementary material available at 10.1186/s13643-024-02481-9.

## Strengths and limitations of this study

- We will describe and summarize existing neuroscientific evidence on human empathy for pleasure.
- The “empathy for pleasure” umbrella term will be used to group various related concepts, such as positive empathy, vicarious joy, empathic joy, and empathic happiness.
- By utilizing a three-factor categorization in our data analysis step, we plan to systematize this field of study.
- Our description and categorization of neuroscientific studies on human empathy for pleasure might not be exhaustive.

## Background

Empathy, the ability to vicariously share and understand others’ emotions and sensations, is quintessential to social species, including humans [4, 5, 18]. The past couple of decades have witnessed an exponential growth of neuroscientific studies on this topic, originating not only from a pure interest in comprehending what makes us a well-functioning social species, but also in examining the behavioral consequences and neural underpinnings of sharing others’ emotions, and how this might be applied for making this world a better place [6]. However, most of the neuroscience literature has hitherto focused on the behavioral and neural correlates of empathizing with others’ negative emotions, and in particular pain [7, 13–15]. Focusing on negative emotions provided novel, key findings, yet neglected the other side of the empathy spectrum, *scilicet* empathy for pleasure. In fact, empathizing with others’ pleasurable outcomes is an evolutionarily and socially relevant process [13, 14, 22], and it should be given the appropriate scientific attention. If done systematically, we believe this will benefit not only basic research about how the human mind works but also research aimed at exploring breakdowns of empathy in neuro-psychiatric disorders, which are, to date, mostly focusing on negative empathy.

A momentous attempt to fill the gap is the review by Morelli and colleagues [13, 14], who have set the stage for the investigation of positive empathy, defined as “understanding and vicariously sharing others’ positive emotions” ([13, 14], p. 58). However, as also argued by the authors themselves (*ibidem*), being an extremely recent construct, positive empathy shares theoretical features with other concepts [13, 14]. This is clear when one acknowledges the different terms that have been associated with the sharing of others’ positive emotions or outcomes, such as vicarious reward [11–14], empathic joy [3], and empathic happiness [9, 10, 21]. Moreover, certain types of rewards experienced by others have been seldomly examined from a neuroscientific standpoint — but see [13, 14] for a comprehensive meta-analysis of personal and vicarious reward studies. For instance, it is currently unexplored, and thus unclear, what are the common and distinct features — at the neural level — of empathizing with others’ discrete emotions (e.g., happiness), sensory gratification (e.g., pleasant touch), or other incentives (e.g., monetary and social rewards). While studies have provided early evidence of the neural correlates of some of these processes, the heterogeneity of conceptualizations and the disconnectedness among the various sources hurdled the emergence of a coherent field of study. At this stage then, it is necessary to assess the type of available evidence to try and clarify working definitions and conceptual boundaries of the topic. Importantly, we try and do this by introducing the concept of empathy for pleasure as an umbrella construct that comprises instances where an individual empathizes with a target’s emotion, feeling, or outcome that is evaluated to be pleasant for them.

Tricco and colleagues [24] state that, differently from systematic reviews, scoping reviews “are used to present a broad overview of the evidence pertaining to a topic, irrespective of study quality, and are useful when examining areas that are emerging, to clarify key concepts and identify gaps” ([24], p. 2). Thus, we believe this knowledge synthesis approach to be the most appropriate to examine, summarize, and describe the available evidence on the potentially crucial topic of empathy for pleasure that, however, has to be considered still in its infancy. We plan to map the foundational concepts of this research area and the main sources and types of evidence available, thus allowing future researchers to frame their work on the topic with the aim to define the relevant theoretical and conceptual backgrounds.

A preliminary search of Scopus, MEDLINE, and the Cochrane Database of Systematic Reviews was conducted on the 29th of December 2022 by M. Mello. In this preliminary search, no current or ongoing scoping reviews on the same subject were identified.

## Review questions

According to Arksey and O’Malley [1], the first stage when conducting a scoping review (as well as other types of knowledge synthesis) is to define the review (research) questions, as this is essential when considering what kind of search strategies to implement. Based on the aims stated in the Introduction, the following review questions will be addressed:

1. What type of neuroscientific evidence about human empathy for pleasure is available?
2. Can we easily clarify working definitions and conceptual boundaries of this research field?
3. What are, at the neural level, the common features of empathizing with different types of rewards?
4. What are, at the neural level, the distinct features of empathizing with different types of rewards?

## Eligibility criteria

Eligibility criteria were established by referring to the population, concept, and context framework. Studies will be selected in accordance with the following criteria.

### Participants

This scoping review is intended to cover studies on empathy for positive emotions, pleasant sensory gratifications, and other rewards in healthy individuals, i.e., with no formal diagnosis of any neurological and/or psychiatric condition, and in people affected by neuropsychiatric and neurodevelopmental disorders. When the available literature will allow us to do so, we will focus our discussion on specific subgroups of conditions. Evidence on all age groups will be included and discussed separately in our scoping review.

### Concept

The concepts to be explored in the present scoping review include positive empathy, vicarious reward, empathic joy, empathic happiness, and empathy for pleasure. This scoping review will not take into consideration studies on empathy for negative emotions and conditions, as well as empathy for anxiety [20] and similar feelings. Furthermore, we will exclude studies focusing on concepts that are related to, but not coincident with, empathy for pleasure, such as positive affect, warm glow, and perceived positive empathy [13, 14].

### Context

This review will consider studies conducted in any context and geographical location.

### Types of sources

This scoping review will mainly consider peer-reviewed experimental studies within the fields of cognitive, affective, and social neuroscience in healthy and neurological and neuropsychiatric patients. Moreover, it will consider conference articles only when peer-reviewed. As one of the main aims of this scoping review is to shed light on the neural correlates of empathy for pleasure, it will focus on works based on functional neuroimaging techniques like functional magnetic resonance imaging (fMRI) and electro-/magnetoencephalography (EEG/MEG), but it will also consider non-invasive brain stimulation studies (e.g., based on transcranial magnetic stimulation, TMS, transcranial electrical stimulation, TES, focal ultrasound stimulation, FUS). In addition, systematic reviews/meta-analyses that meet the inclusion criteria will also be scrutinized for useful evidence, depending on their research questions.

## Methods

The present review will follow the JBI methodology for scoping reviews [16, 17]. It was designed and will be conducted in accordance with the PRISMA-ScR (Preferred Reporting Items for Systematic Reviews and Meta-Analyses extension for Scoping Reviews) framework [23]. The objectives, inclusion criteria, and methods for this scoping review were specified in advance and documented on Open Sciences Framework registries 10.17605/OSF.IO/W7H6J.

## Search strategy

An initial limited search of Scopus was undertaken to identify articles on the topic. The text words contained in the titles and abstracts of relevant articles, and the index terms used to describe the articles were used to develop a full search strategy (see Table 1 in Appendix 1). This consisted of two main parts joined together by Boolean operators: the first restricted the search to studies on empathy and vicarious experiences; the second focused on positive experiences (using all the related terms we found in the initial search and/or we knew were relevant). Thus, our strategy considered synonyms and related terms, used Boolean operators, and explored Medical Subject Headings (MeSH) terms. The search strategy, including all identified keywords and index terms, will be adapted for each included database and/or information source. The reference list of all included sources of evidence will be screened for additional studies. Boolean operators (i.e., “OR” and “AND”) will be used to combine and refine search terms and concepts.

Studies published in English since January 2000 will be included. The databases to be searched include Scopus, MEDLINE (PubMed), ScienceDirect, and Web of Science.

### Study/source of evidence selection

Following the search, all identified citations will be collated and uploaded into Zotero and duplicates removed. Following a pilot test, titles and abstracts will then be screened by M. Mello for assessment against the inclusion criteria for the review.

Following this initial evaluation of all citations, a second assessment will be carried out by M. Mello and M. Fusaro. Screening differences will be resolved between the two researchers, and in cases where an agreement cannot be reached, a senior researcher will be consulted (S. M. Aglioti). Potentially relevant sources will be retrieved in full, and their citation details imported into Zotero. The full text of selected citations will be assessed in detail against the inclusion criteria by the two independent reviewers. Reasons for exclusion of sources of evidence at full text that do not meet the inclusion criteria will be recorded and reported in the scoping review. Any disagreements that arise between the search products assessors at each stage of the selection process will be resolved through discussion, or with one or more additional assessor/s. The results of the search and the study inclusion process will be reported in full in the final scoping review.

The results of the search will be reported in full and presented in a PRISMA-ScR flow diagram [23].

### Data extraction

Data will be extracted from papers included in the scoping review by two independent assessors using a data extraction tool developed by the reviewers. The extracted data will include some standard information (such as author/s, year of publication, study objectives) as well as specific details about the participants, concepts, and context. To this aim, a data charting table will be developed and piloted at the protocol stage. A draft of this tool is provided in Table 2 in Appendix 2, and it consists of minor revisions to the original JBI template [17].

Moreover, we will categorize the extracted data according to three main factors:

1. Type of “pleasure” the study participants empathize with:
  - Social/affiliative reward
  - Positive affect
  - Sensory event
  - Monetary reward
2. Type of neuroscientific methodology utilized
  - fMRI/functional near-infrared spectroscopy (fNIRS)
  - EEG/MEG
  - Non-invasive brain stimulation (TMS, TES, FUS)
  - Intracranial brain stimulation
3. Type of study sample
  - Healthy participants
  - People affected by neurological disorders
  - People affected by psychiatric disorders
  - People with neurodevelopmental disorders

This categorization will be included in a separate table (see Table 3 in Appendix 3). The draft data charting tools will be modified and revised as necessary during the process of extracting data from each included evidence source. Modifications will be detailed in the scoping review. Any disagreements that may arise between the assessors will be resolved through discussion, or by enlisting the advice of one or more additional assessor/s. If appropriate, authors of papers will be contacted to request missing or additional data, where required.

### Data analysis and presentation

The primary aims of a scoping review are mapping the key concepts underlying a field of study, clarifying its working definitions and conceptual boundaries, and ultimately providing an overview of the available evidence. Based on this, the analysis of the extracted data will consist in aggregating, qualitatively evaluating, and describing the findings of the included studies. Thematic analysis will be conducted to provide an overview of qualitative data relating to the types of pleasant outcome the study participants empathize with and the type of neuroscientific methodology utilized.

The extracted and analyzed data will be presented in a tabular/graphical format that is congruent with the scoping review’s proposed research questions. The tabulated or charted data will be accompanied by a summary, a synthesis, and a discussion of the findings.

The full scoping review will be reported in accordance with the PRISMA-ScR checklist (Additional file 1) [23].

## Discussion

Empathizing with others’ negative emotions is extremely important for social species. Research on empathy for negative emotions, particularly for pain, has resulted in an abundance of studies on the topic [4]. In contrast, the examination of the cognitive and neural mechanisms of sharing and understanding others’ pleasures has been neglected. There is, however, no scientific or theoretical reason why empathizing with others’ positive emotions is not important for interpersonal functioning [19]. In fact, early evidence suggests that also positive empathy is highly relevant from an evolutionary and social point of view, as it is associated with increased positive affect, well-being, and prosocial behavior [13, 14, 22]. For instance, Morelli and colleagues [12] found that sensitivity to personal reward or reward to a close friend correlated with individuals’ psychological well-being. Particularly interesting, from a social neuroscience perspective, is the link between positive empathy and prosocial behavior: as Telle and Pfister [22] describe, the positive affect experience originating from sharing others’ positive emotions, that people generally desire to maintain, can promote prosocial behavior, which, in turn, serves to preserve the positive affective state [22]. 

This scoping review will provide an overview of the available neuroscientific evidence on human empathy for pleasure. The aims we set out to achieve include mapping the key concepts underlying this emergent field of research and clarifying its working definitions and conceptual boundaries. We will do this by providing an overview of the existing evidence and by answering questions regarding its heterogeneity and, ultimately, by emphasizing the gaps in the literature on this topic.

Thus, we will hopefully provide new insights on this field of research, sparking researchers’ interest and informing future studies.

### Supplementary Information

**Additional file 1.** PRISMA checklist.

## Data Availability

Not applicable.

## Appendix 1

**Table 1:** Search strategy

**Database**	**Search strategy**	***N*** **°**
SCOPUS	(TITLE-ABS-KEY(empath* OR vicarious) AND TITLE-ABS-KEY(pleas* OR joy OR reward OR positive OR happ*)) AND PUBYEAR > 1999 AND (LIMIT-TO(SUBJAREA, “MEDI”) OR LIMIT-TO(SUBJAREA, “PSYC”) OR LIMIT-TO (SUBJAREA, “SOCI”) OR LIMIT-TO(SUBJAREA, “NEUR”)) AND (LIMIT-TO(LANGUAGE, “English”))	7693
MEDLINE (PubMed)	(((empath*[Title/Abstract] OR vicarious[Title/Abstract]) OR (empath*[MeSH Terms])) AND ((pleas*[Title/Abstract] OR pleas*[MeSH Terms]) OR (joy[Title/Abstract] OR joy[MeSH Terms]) OR (reward[Title/Abstract] OR reward[MeSH Terms]) OR (positive[Title/Abstract] OR positive[MeSH Terms]) OR (happ*[Title/Abstract] OR happ*[MeSH Terms]))) AND English[Language] AND (2000:2023[pdat])	4918
Web of Science	ALL = (empath* OR vicarious) AND ALL = (pleas* OR joy OR happ* OR positive OR reward) Year = 2000–2023	9164

## Appendix 2

**Table 2:** Data extraction instrument — basic information

**Scoping review details**	
Scoping review title:	
Review objective/s:	
Review question/s	
**Inclusion/exclusion criteria**	
Population	
Concept	
Context	
Types of study	
**Evidence source details and characteristics**	
Study citation details (e.g., author/s, date, title, journal, volume, issue, pages)	
Country	
Context	
Participants (details e.g., age/sex and number)	

## Appendix 3

**Table 3:** Data extraction instrument — three-factor categorization

**Publication**	**Type of pleasant outcome**	**Type of neuroscientific methodology**	**Sample**
e. g., Lamm et al. [8]	Sensory outcome (pleasant touch)	fMRI	Healthy
e. g., Balconi and Vanutelli [2]	Social/affiliative reward	EEG/fNIRS	Healthy

## Abbreviations

WoS: Web of Science
fMRI: Functional magnetic resonance imaging
EEG: Electroencephalography
MEG: Magnetoencephalography
TMS: Transcranial magnetic stimulation
TES: Transcranial electrical stimulation
FUS: Focal ultrasound stimulation
